# Above-Threshold Queries of Environmental Conditions Based on Bilinear Interpolation in Wireless Sensor Networks

**DOI:** 10.3390/s18124203

**Published:** 2018-11-30

**Authors:** Yaxi Liu, Wei Huangfu, Haijun Zhang, Keping Long

**Affiliations:** Beijing Advanced Innovation Center for Materials Genome Engineering, Beijing Engineering and Technology Research Center for Convergence Networks and Ubiquitous Services, School of Computer and Communication Engineering, University of Science and Technology Beijing(USTB), Beijing 100083, China; b20160295@xs.ustb.edu.cn (Y.L.); haijunzhang@ieee.org (H.Z.); longkeping@ustb.edu.cn (K.L.)

**Keywords:** wireless sensor networks, spatial database queries, window queries, above-threshold queries, bilinear interpolation

## Abstract

Wireless sensor networks can be regarded as sensor database systems, which permit users to query sensor data of interest. Among various spatial database queries, we focus the area-wise aggregate queries in the region where the sensor values are above a predefined threshold, which are summarized as above-threshold queries. In this paper, we propose a novel Bilinear Interpolation-Based (BIB) algorithm, which utilizes the bilinear interpolation to estimate the environmental variables inside a grid with the known sensor values at the vertexes, to support the above-threshold queries for regularly-deployed sensor networks and provide the closed-form solution of the above-threshold ratio. We designate experiments with both the artificially-constructed environment data and the real temperature data. Experiment results manifest that the proposed BIB algorithm shows a good performance in estimating the above-threshold ratios to support the above-threshold queries in an accurate and efficient manner.

## 1. Introduction

Wireless sensor networks are sharply emerging as one of the most significant technologies to bridge the gap between the physical world and the digital world [1,2,3]. In order to sense spatial natural phenomena in the physical world remotely, wireless sensor networks usually consist of many smart energy-efficient devices, namely sensor nodes that are equipped with inexpensive embedded sensors, processors, memories, radio communication modules, and power supplies to gather environmental conditions, ranging from temperature, precipitation, smoke, soil humidity to atmospheric pressure, to name a few. Wireless sensor networks provide us prolific spatiotemporal environment data and will play important roles in many applications, including environmental monitoring, target tracking, precision agriculture, and so forth [4,5,6,7,8,9].

Since data are constantly being sent from sensors, wireless sensor networks can be regarded as sensor database systems, such as TinyDB [10]. The ability to apply various kinds of data-querying techniques to sensors and process data is highly desirable. Various methods and techniques of data storage and data query have been proposed, which permit users to manage sensor data of interest [11,12].

Spatial database queries, which gather environmental variables, from the perspective of geography, inside a particular geographical region of interest (ROI), have become one of the primary technologies due to the spatial characteristics of the collected environmental conditions [13,14]. As one of the various kinds of spatial database queries, window queries mainly refer to those that retrieve the sensor data inside a specified geographical window, e.g., a two-dimensional rectangular region $\left\{\left(X,Y\right)|X_{min}\leq X\leq X_{max},Y_{min}\leq Y\leq Y_{max}\right\}$ [15]. Some complicated spatial queries are used to determine the region in which the sensor values satisfy a predefined condition [16,17,18]. For example, in Ref. [19], in order to find out the relationships between the weather and the habitats of animal species, scientists should query the species densities in the rainy regions and those in the sunny regions. Assume that when the humidity function H(X,Y) is above the rainy threshold ${\mathrm{TH}}_{\mathrm{humidity}}$, it begins to rain, and vice versa. Thus, the queries should determine the region where the sensor values are above a given threshold from humidity sensors, denoted as $\left\{\left(X,Y\right)|H\left(X,Y\right)\geq {\mathrm{TH}}_{\mathrm{humidity}}\right\}$.

Aggregate queries, another critical type of database query, refer to the summaries of the current sensor values by implementing the basic database aggregates such as COUNT, MIN, MAX, SUM, AVERAGE, etc. [20,21]. Queries are often combined with aggregation operations to support complex analysis of environmental data [19,22]. Due to the spatial characteristics of the environmental conditions, queries should support the area-wise aggregate operation. In Ref. [23], Aslan proposed a forest fire detection and monitoring framework. In such a case, we will pay more attention to the total area where the temperature values are above a predefined threshold, i.e., the above-threshold area. An alarm should be triggered, for example, if the area of the hot subregion where the temperature is above 40 ${}^{\circ }C$ is greater than 5% of the total region. Here, the query will retrieve the area $\left|\{(X,Y)|T(X,Y)\geq 40\;{}^{\circ }C\}\right|$, and we will run the aggregate query on the spatial region determined by the sensor values, like “SELECT AREA(*) from temperature_table where T > 40${}^{\prime \prime }$. We summarize such area-wise aggregate queries in the region where the sensor values are above a predefined threshold as above-threshold queries in this paper.

Furthermore, there are two deployment patterns in sensor networks to achieve particular environmental monitoring: random deployment and the regular (deterministic) one [24]. Although the regular deployment scheme is considered to be too ideal to implement in practical applications, it usually has better network coverage and connectivity than the random deployment [24,25,26]. Furthermore, it is practically developed in some applications such as forest fire prevention and high temperature alert [23] and offers us a basis to further our research. Thus, we will only focus on the regular deployment for wireless sensor networks.

For the regular deployment of wireless sensor networks, there are some related techniques to support the above-threshold queries. The Nearest Neighbor (NN) algorithm is a technique to estimate the environmental conditions only by the sensor data provided by the nearest sensors, which leads to a Voronoi diagram [27], to enable the accumulation of the areas of polygons, inside which the estimated environmental conditions are beyond the threshold. The Marching Square (MS) algorithm is a computer graphics algorithm to generate contours for a two-dimensional scalar field, referring to environmental conditions here, and thus also supports the above-threshold queries based on the isobars [28,29]. Both algorithms are extensively studied. However, the NN algorithm, only taking the information about whether the sensor values are greater than the threshold or not into account, is robust, but inaccurate. The MS algorithm performs the linear interpolation to estimate the environmental conditions on the edges based on the sensor values, which usually produces more accurate results. Nevertheless, the MS algorithm determines the above-threshold subregion by a straightforward connection of all the points on the edges by interpolation, and thus, the environmental conditions inside the region are not adequately considered.

In this paper, we propose a novel Bilinear Interpolation-Based (BIB) algorithm to support the above-threshold queries for regularly-deployed sensor networks. We utilize the bilinear interpolation to estimate the environmental variables inside a square with the known sensor values from sensor nodes located at its four vertexes. The information of environmental variables is taken into full consideration by performing interpolation inside the grid. Thus, the area of the above-threshold subregion can be obtained by mathematical formulation, and finally, we propose the closed-form solution of the above-threshold ratio. In order to evaluate the performance of different above-threshold query algorithms, we first use the Kriging interpolation, which can be regarded as the gold standard to compare the interpolation weights of various algorithms. The results of theory analysis show that the proposed BIB above-threshold algorithm is an approximation of the Kriging algorithm, while the NN algorithm has a large calculation error in geographic data analysis. Then, we perform the artificially-constructed environment data experiment and real temperature data experiment to draw the boundaries and check the metrics of False Positives (FP), False Negatives (FN), and Mean Squared Error (MSE). Experiment results show that the proposed BIB algorithm has a good performance in estimating the above-threshold ratios to support the above-threshold queries in an accurate and efficient manner.

The rest of the paper is organized as follows. Section 2 discusses related works on the applications of the regular deployment sensor networks and enumerates the database management techniques for window queries and aggregate queries. Section 3 presents the system model and formulates the problem. Section 4 introduces the bilinear interpolation-based above-threshold query algorithm. Section 5 describes the experiments and discusses the results. Finally, Section 6 concludes this paper.

## 2. Related Work

Quite extensive distributed database management techniques have been proposed to support the spatial queries, mainly the window queries. Berchtold et al. [30] proposed a pyramid technique to index data in the high-dimensional database system, which is efficient for window queries. In Ref. [31], Ooi et al. presented another tunable index scheme called iMinMaxfor high-dimensional window queries, which was less complex and performed better than the pyramid technique. However, there are two major issues impeding the window queries: the lack of explicit window semantics and the low implementation efficiency. To solve this problem, Li et al. [22] established a window query technique framework by defining window semantics to avoid the intra-operator buffers and the reprocessing tuples. However, the previous works have not yet explored the spatial property in sensor network databases. Xu et al. [32] investigated a framework of spatial window query in dynamic sensor networks. To further simplify the query process, Xu et al. [33] proposed an infrastructure-free window query technique in sensor networks. Ku et al. [34] presented an approach to reduce the access latency of the spatial queries, e.g., window queries, by utilizing the neighbor information in wireless broadcast environments. The geographical location information can guide the window queries due to the spatial correlation, and the inspiration of the spatial-aware approach can also be used in wireless sensor networks. The existing database management techniques mainly focus on the geographical coordinate-type window queries, i.e., the traditional window queries, rather than the spatial region determined by environmental values. The environmental value-determined spatial region was also studied [19,23]. Note that the window queries are not only restricted to the spatial coordinators, but also applied to the environmental conditions. The latter window queries to retrieve the sensor values inside a predefined interval were discussed [16,17,18]. It is obvious that such queries degenerate into above-threshold ones if the lower bound of the query window is the threshold and the upper bound is assigned to the positive infinite.

The spatial queries combined with aggregate operation, which are closely related to the above-threshold query techniques in this paper, have also been studied and used in some environmental monitoring applications. In order to figure out the influence factors of climate changes, Wang et al. [35] established a window query algorithm to support various aggregate queries on three-dimensional time-varying datasets, such as the AVERAGE, MIN, and MAX operations.

The deployment is significant for sensor networks. A regular deployment pattern is important in some scenarios such as disaster alert [24,25,26]. In Ref. [36], Bulusu proposed a GPS (Global Positioning System)-less low-cost outdoor localization model for small devices, e.g., sensors, which are placed in a regular mesh structure. Wang et al. [37] proposed a deployment algorithm in an arbitrarily-shaped region and used the regular sensor patterns in a region with boundaries and obstacles. In Ref. [38], the typical regularly-deployed oilfield with an area of 8 × 8 km required approximately a 50–100 m distance between the sensors in seismic analysis. In Ref. [23], Aslan proposed a forest fire detection and monitoring framework and implemented it in regularly-deployed wireless sensor networks. Two popular layout models—square layout, i.e., temperature sensors are placed at the corners of squares, and hexagonal layout, i.e., sensors are placed at the corners of the hexagons—were adopted.

To estimate the environmental conditions at a given location to support spatial queries, the NN and MS algorithms are usually used. The former estimates the environmental conditions only by the nearest sensors, while the latter generates the isobars by the linear interpolation on the edges. Both are applicable to the above-threshold queries. The NN algorithm is simple, and its computation workload and local communication traffic load are small. The MS algorithm is more accurate by taking the environmental conditions on the edges into account. However, neither adequately exploits the sensor values to better the interpolation of the environmental conditions inside the region.

Thus, the idea to fully exploit the sensor values to better the interpolation inside the region inspires us to propose a novel BIB algorithm for the above-threshold queries. With small incrementation of the computation workload and the local communication traffic, the proposed BIB algorithm performs interpolation inside the region and deduces a closed-form above-threshold query solution. It is not only more accurate than the NN and MS algorithms, but also accordant with the gold standard in the geographical sense.

## 3. System Model and Problem Formulation

In this section, we present the system model and formulate the problem.

### 3.1. System Model

Consider a wireless sensor network deployed in a predefined square region *R*, consisting of *N* sensor nodes, $e_{1},e_{2},\ldots ,e_{N}$. Each sensor node senses and then transfers the environment condition data to the database server. Let f(x,y) be the environmental variable at the position with the coordinate (x,y). For a given threshold *T*, the above-threshold (sub-)region query is used to evaluate the above-threshold region $R_{T}$, which is defined as:

(1) $$R_{T}≜\left\{\left(x,y\right)|f\left(x,y\right)\geq T,\left(x,y\right)\in R\right\}.$$

The above-threshold ratio is then defined as:

(2) $$C_{T}≜\frac{\left|R_{T}\right|}{\left|R\right|}$$

where $\left|X\right|$ is the cardinality, i.e., the area of the region *X*.

**Figure 1:**
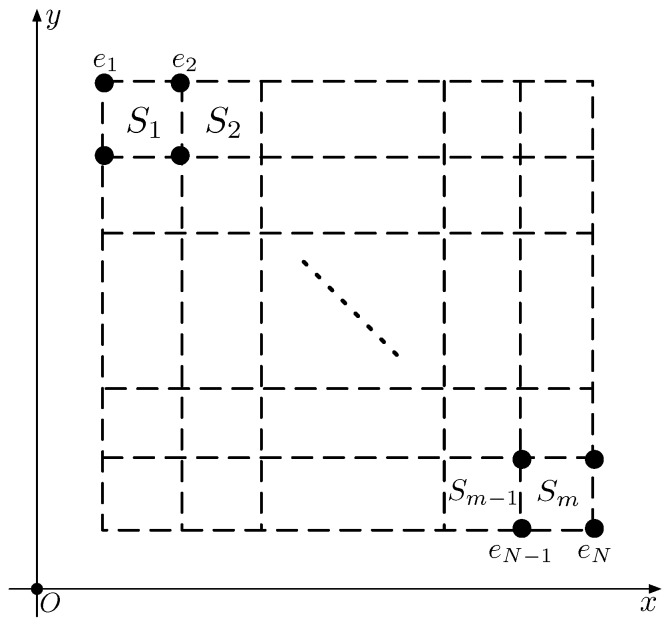
Illustration of the system.

Suppose that the region *R* is split into many regular non-overlapping rectangle grids, as depicted in Figure 1. All the rectangle subregions are listed as $S=\left\{S_{1},S_{2},\ldots ,S_{m}\right\}$, where *m* is the total number of rectangle subregions.

There are four sensor nodes distributed at four vertexes of $S_{i}$. Sensors can gather environmental variables, which can be used to estimate the above-threshold ratio. Notice that adjacent rectangles may share some sensor nodes.

Then, the area-wise above-threshold ratio is given by:

(3) $$C_{T}=\frac{\sum_{i=1}^{m}\left|S_{Ti}\right|}{\sum_{i=1}^{m}\left|S_{i}\right|}=\frac{\sum_{i=1}^{m}\left|S_{Ti}\right|}{\left|R\right|}$$

where $S_{Ti}$ is defined as

(4) $$S_{Ti}≜\left\{\left(x,y\right)|f\left(x,y\right)\geq T,\left(x,y\right)\in S_{i}\right\}.$$

### 3.2. Problem Formulation

Our goal is to estimate the above-threshold ratio $C_{T}$. From Equation (3), we can obtain $C_{T}$ by summing the above-threshold area limited inside each $S_{i}$. Thus, the next objective is to calculate $\left|S_{Ti}\right|$ according to the coordinates and the sensor values of the four vertexes in $S_{i}$.

**Figure 2:**
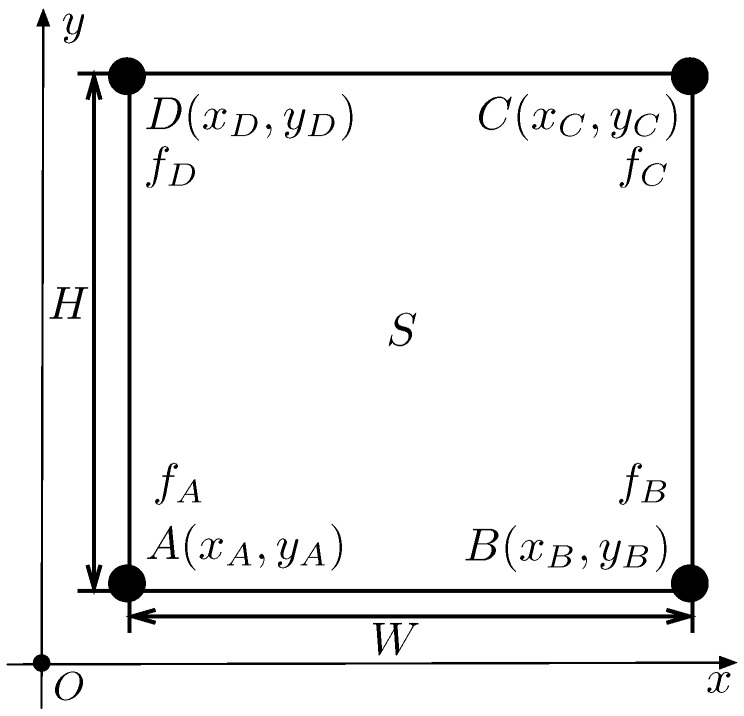
Illustration of rectangle subregion characteristics.

Without loss of generality and for the sake of simplicity, we only consider one of the rectangle subregions and rewrite such a region $S_{i}$ as *S*. The vertex coordinates of rectangle *S* are $A(x_{A},y_{A})$, $B(x_{B},y_{B})$, $C(x_{C},y_{C})$, and $D(x_{D},y_{D})$, and the sensor values at four vertexes are $f_{A}$, $f_{B}$, $f_{C}$, and $f_{D}$, respectively. Let *W* and *H* be the length and the height of *S*, as shown in Figure 2.

Now, the goal is to calculate the above-threshold area inside *S* with length *W* and height *H* under the condition of known coordinates and the sensor values of four vertexes. The above-threshold region $S_{T}$ in *S* is defined as:

(5) $$S_{T}≜\left\{\left(x,y\right)|f\left(x,y\right)\geq T,\left(x,y\right)\in S\right\}.$$

## 4. Bilinear Interpolation-Based Above-Threshold Query Algorithm

In this section, we propose the bilinear interpolation-based algorithm and describe the nearest neighbor algorithm and the marching square algorithm. Furthermore, we introduce the evaluative metrics of mean squared error, false positive, and false negative.

### 4.1. Coordinate Transformation and Boundary of the Above-Threshold Region

In order to simplify the calculation, we perform a translation and an affine transformation to convert the coordinate (x,y) inside *S* to $(u=\frac{x-x_{A}}{W}$, $v=\frac{y-y_{A}}{H})$. Now, the region *S* is converted into a unit square with $u_{A}=0$ and $v_{A}=0$. Thus, we have $S=\left\{(u,v)|0\leq u\leq 1,0\leq v\leq 1\right\}$. To obtain the original above-threshold area, the result in this subsection should be multiplied by W·H due to the area unit dx·dy=W·H·du·dv.

We utilize the bilinear interpolation to estimate the environmental variables inside *S*. Bilinear interpolation is a widespread interpolation algorithm for two variables on a two-dimensional grid. It is an extension of linear interpolation for one variable. The key idea of bilinear interpolation is to perform linear interpolation first in one direction and then again in the other direction. Although each step is linear, the bilinear interpolation as a whole is not linear, but rather, quadratic.

The sensor value at arbitrary point (u,v) inside *S* can be interpolated by the sensor values of four vertexes based on bilinear interpolation. Denote $\hat{f}\left(u,v\right)$ as the estimated environmental variable. Thus,

(6) $$\begin{array}{cc} \hat{f}\left(u,0\right)= & \left(1-u\right)f_{A}+uf_{B},\\ \hat{f}\left(u,1\right)= & \left(1-u\right)f_{D}+uf_{C},\\ \hat{f}\left(u,v\right)= & \left(1-v\right)\hat{f}\left(u,0\right)+v\hat{f}\left(u,1\right)\\ = & a_{11}uv+a_{10}u+a_{01}v+a_{00} \end{array}$$

where:

(7) $$\begin{array}{cc} a_{11}= & f_{A}+f_{C}-f_{B}-f_{D},\\ a_{10}= & -f_{A}+f_{B},\\ a_{01}= & -f_{A}+f_{D},\\ a_{00}= & f_{A}. \end{array}$$

For the four-vertex interpolation in $S=\left\{(u,v)|0\leq u\leq 1,0\leq v\leq 1\right\}$, the estimated sensor value of arbitrary coordinate (u,v) in the square is given by:

(8) $$\hat{f}\left(u,v\right)=W^{T}\left(u,v\right)\cdot \left(\begin{array}{c} f_{A}\\ f_{B}\\ f_{C}\\ f_{D} \end{array}\right)$$

where W(u,v) is a 4×1 matrix, which implies the interpolation weights of four vertexes. We can conclude from Equations (6) and (7) that:

(9) $$W_{\mathrm{BIB}}\left(u,v\right)=\left(\begin{array}{c} uv-u-v+1\\ u-uv\\ uv\\ v-uv \end{array}\right).$$

Denote the estimated above-threshold region ${\hat{S}}_{T}$ as $\left\{\left(u,v\right)|\hat{f}\left(u,v\right)\geq T,\left(u,v\right)\in S\right\}$ and the estimated above-threshold ratio ${\hat{C}}_{T}$ as $\frac{1}{\left|R\right|}\cdot \sum_{i=1}^{m}\left|{\hat{S}}_{Ti}\right|$. The boundary of ${\hat{S}}_{T}$, which lies between the above-threshold part and the below-threshold one, is given by:

(10) $$\hat{f}\left(u,v\right)=a_{11}uv+a_{10}u+a_{01}v+a_{00}=T.$$

**Figure 3:**
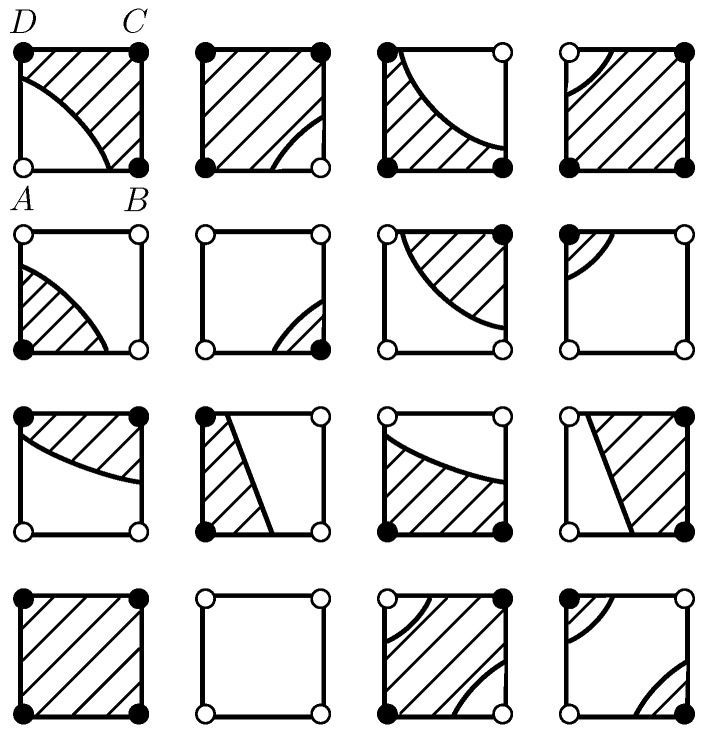
Illustrations of the boundaries and the above-threshold regions with the Bilinear Interpolation-Based (BIB) algorithm.

The boundary $\hat{f}\left(u,v\right)=T$ is hyperbolic if $a_{11}\neq 0$ and is degraded to a straight line if $a_{11}=0$. Various situations for the boundaries and the above-threshold regions with the BIB algorithm are shown in Figure 3. The solid black circles on the vertexes represent the above-threshold values, and the hollow ones represent the below-threshold values. The threshold *T* is assigned as zero. The black lines are the boundaries where $\hat{f}\left(u,v\right)=T$, and the shaded areas are the above-threshold regions where $\hat{f}\left(u,v\right)\geq T$. In such a concrete case, the sensor values at points *A*, *B*, *C*, and *D* are assigned to ±3, ±2, ±4, and ±1, respectively, depending on whether the sensor values are above or below the threshold.

### 4.2. Above-Threshold Area and Above-Threshold Ratio

The above-threshold area $|{\hat{S}}_{T}|$ can be discussed for different cases. If the boundary is hyperbolic ($a_{11}\neq 0$), the expression of $\hat{f}\left(u,v\right)$ can be transformed to:

(11) $$\hat{f}\left(u,v\right)=a_{11}\hat{u}\hat{v}+Q$$

where:

(12) $$\begin{array}{c} \hat{u}=u+\frac{a_{01}}{a_{11}}, \end{array}$$

(13) $$\begin{array}{c} \hat{v}=v+\frac{a_{10}}{a_{11}}, \end{array}$$

(14) $$\begin{array}{c} Q=\frac{a_{00}a_{11}-a_{01}a_{10}}{a_{11}}. \end{array}$$

The boundary $\hat{f}\left(u,v\right)=a_{11}\hat{u}\hat{v}+Q=T$ is equivalent to $\hat{u}\hat{v}=E$ where $E=\frac{T-Q}{a_{11}}$. Let $G_{E}$ denote the region $\hat{u}\hat{v}\geq E$, defined as:

(15) $$G_{E}=\left\{\left(\hat{u},\hat{v}\right)|\hat{u}\hat{v}\geq E\right\}.$$

**Figure 4:**
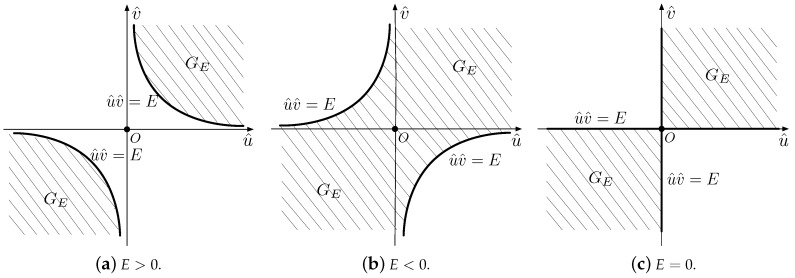
Illustrations of the boundaries and the regions $G_{E}$ in the $\hat{u}o\hat{v}$ plane if $a_{11}>0$.

If $a_{11}>0$, ${\hat{S}}_{T}$ is the intersection of the region $G_{E}$ and the region $\hat{S}$, or precisely, ${\hat{S}}_{T}=\hat{S}\cap G_{E}$. Otherwise, if $a_{11}<0$, we have ${\hat{S}}_{T}=\hat{S}\backslash G_{E}$. Without loss of generality, we only discuss the case of $a_{11}>0$. The illustrations of the boundaries and the above-threshold regions in the $\hat{u}o\hat{v}$ plane if $a_{11}>0$ are shown in Figure 4.

We denote an auxiliary function $k(E,{\hat{u}}_{0},{\hat{v}}_{0})$ as

(16) $$k\left(E,{\hat{u}}_{0},{\hat{v}}_{0}\right)=\int_{0}^{{\hat{u}}_{0}}\int_{0}^{{\hat{v}}_{0}}{𝟙}_{G_{E}}\left(\hat{u},\hat{v}\right)\;d\hat{u}\;d\hat{v}$$

where ${𝟙}_{X}\left(u,v\right)$ is the characteristic function of the set X, which satisfies:

(17) $${𝟙}_{X}\left(u,v\right)=\left\{\begin{array}{cc} 1 & if\;(u,v)\in X,\\ 0 & otherwise. \end{array}\right.$$

Furthermore, let us define:

(18) $$\begin{array}{cc} U= & \left\{\begin{array}{cc} \left[0,{\hat{u}}_{0}\right] & {\hat{u}}_{0}\geq 0,\\ {}[{\hat{u}}_{0},0] & {\hat{u}}_{0}<0, \end{array}\right. \end{array}$$

(19) $$\begin{array}{cc} V= & \left\{\begin{array}{cc} \left[0,{\hat{v}}_{0}\right] & {\hat{v}}_{0}\geq 0,\\ {}[{\hat{v}}_{0},0] & {\hat{v}}_{0}<0. \end{array}\right. \end{array}$$

Denote the symbol “+” as the positive sign of *E*, ${\hat{u}}_{0}$, or ${\hat{v}}_{0}$ and the symbol “−” as the negative one. For example, the notation “+++” of the symbol $k^{+++}\left(E,{\hat{u}}_{0},{\hat{v}}_{0}\right)$ indicates that all the independent variables *E*, ${\hat{u}}_{0}$, and ${\hat{v}}_{0}$ are positive.

**Figure 5:**
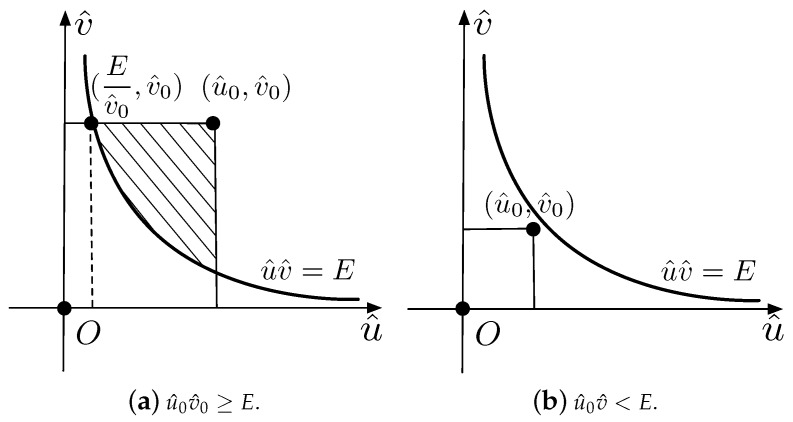
Illustrations of function values $k^{+++}\left(E,{\hat{u}}_{0},{\hat{v}}_{0}\right)$ if E>0, ${\hat{u}}_{0}>0$, and ${\hat{v}}_{0}>0$.

There are two situations if E>0, ${\hat{u}}_{0}>0$ and ${\hat{v}}_{0}>0$. If ${\hat{u}}_{0}{\hat{v}}_{0}\geq E$, the function value $k^{+++}\left(E,{\hat{u}}_{0},{\hat{v}}_{0}\right)=\left|G_{E}\cap \left(U\times V\right)\right|$ is the area of the shaded region shown in Figure 5a. Otherwise, the function value $k^{+++}\left(E,{\hat{u}}_{0},{\hat{v}}_{0}\right)=0$, as shown in Figure 5b.

Thus, we have:

(20) $$k^{+++}\left(E,{\hat{u}}_{0},{\hat{v}}_{0}\right)=\int_{0}^{{\hat{u}}_{0}}\int_{0}^{{\hat{v}}_{0}}{𝟙}_{G_{E}}\left(\hat{u},\hat{v}\right)\;d\hat{u}\;d\hat{v}=\left\{\begin{array}{cc} {\hat{u}}_{0}{\hat{v}}_{0}-E-Eln\frac{{\hat{u}}_{0}{\hat{v}}_{0}}{E} & {\hat{u}}_{0}{\hat{v}}_{0}\geq E,\\ 0 & {\hat{u}}_{0}{\hat{v}}_{0}<E. \end{array}\right.$$

Similarly, the result of E>0, ${\hat{u}}_{0}<0$, and ${\hat{v}}_{0}<0$ can be calculated by:

(21) $$\begin{array}{c} k^{+--}\left(E,{\hat{u}}_{0},{\hat{v}}_{0}\right)=\int_{{\hat{u}}_{0}}^{0}\int_{{\hat{v}}_{0}}^{0}{𝟙}_{G_{E}}\left(\hat{u},\hat{v}\right)\;d\hat{u}\;d\hat{v}=\left\{\begin{array}{cc} {\hat{u}}_{0}{\hat{v}}_{0}-E-Eln\frac{{\hat{u}}_{0}{\hat{v}}_{0}}{E} & {\hat{u}}_{0}{\hat{v}}_{0}\geq E,\\ 0 & {\hat{u}}_{0}{\hat{v}}_{0}<E. \end{array}\right. \end{array}$$

In the same way, we have:

(22) $$\begin{array}{c} k^{+-+}\left(E,{\hat{u}}_{0},{\hat{v}}_{0}\right)=0, \end{array}$$

(23) $$\begin{array}{c} k^{++-}\left(E,{\hat{u}}_{0},{\hat{v}}_{0}\right)=0. \end{array}$$

So far, all the cases of E>0 have been taken into consideration.

If E<0, ${\hat{u}}_{0}>0$ and ${\hat{v}}_{0}>0$, we always have ${\hat{u}}_{0}{\hat{v}}_{0}>E$, then:

(24) $$k^{-++}\left(E,{\hat{u}}_{0},{\hat{v}}_{0}\right)={\hat{u}}_{0}{\hat{v}}_{0}.$$

Similarly, if E>0, ${\hat{u}}_{0}<0$ and ${\hat{v}}_{0}<0$, we have:

(25) $$k^{---}\left(E,{\hat{u}}_{0},{\hat{v}}_{0}\right)={\hat{u}}_{0}{\hat{v}}_{0}.$$

**Figure 6:**
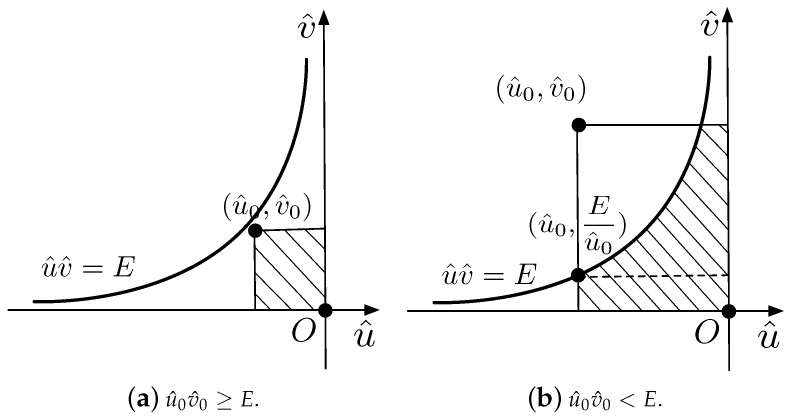
Illustrations of function values $k^{--+}\left(E,{\hat{u}}_{0},{\hat{v}}_{0}\right)$ if E<0, ${\hat{u}}_{0}<0$, and ${\hat{v}}_{0}>0$.

There are two situations for E<0, ${\hat{u}}_{0}<0$ and ${\hat{v}}_{0}>0$. If ${\hat{u}}_{0}{\hat{v}}_{0}\geq E$, the function value $k^{--+}\left(E,{\hat{u}}_{0},{\hat{v}}_{0}\right)={\hat{u}}_{0}{\hat{v}}_{0}$, as shown in Figure 6a. Otherwise, the function value $k^{--+}\left(E,{\hat{u}}_{0},{\hat{v}}_{0}\right)=-\left|U\times V-G_{E}\right|$.

Therefore,

(26) $$\begin{array}{cc} k^{--+}\left(E,{\hat{u}}_{0},{\hat{v}}_{0}\right)= & \int_{0}^{{\hat{u}}_{0}}\int_{0}^{{\hat{v}}_{0}}{𝟙}_{G_{E}}\left(\hat{u},\hat{v}\right)\;d\hat{u}\;d\hat{v}=-\int_{{\hat{u}}_{0}}^{0}\int_{0}^{{\hat{v}}_{0}}{𝟙}_{G_{E}}\left(\hat{u},\hat{v}\right)\;d\hat{u}\;d\hat{v}\\ = & \left\{\begin{array}{cc} -E-Eln\frac{{\hat{u}}_{0}{\hat{v}}_{0}}{E} & {\hat{u}}_{0}{\hat{v}}_{0}\geq E,\\ \hat{u}\hat{v} & {\hat{u}}_{0}{\hat{v}}_{0}<E. \end{array}\right. \end{array}$$

Similarly, the result of E<0, ${\hat{u}}_{0}>0$, and ${\hat{v}}_{0}<0$ is given by:

(27) $$\begin{array}{cc} k^{-+-}\left(E,{\hat{u}}_{0},{\hat{v}}_{0}\right)= & \int_{0}^{{\hat{u}}_{0}}\int_{0}^{{\hat{v}}_{0}}{𝟙}_{G_{E}}\left(\hat{u},\hat{v}\right)\;d\hat{u}\;d\hat{v}=-\int_{0}^{{\hat{u}}_{0}}\int_{{\hat{v}}_{0}}^{0}{𝟙}_{G_{E}}\left(\hat{u},\hat{v}\right)\;d\hat{u}\;d\hat{v}\\ = & \left\{\begin{array}{cc} -E-Eln\frac{{\hat{u}}_{0}{\hat{v}}_{0}}{E} & {\hat{u}}_{0}{\hat{v}}_{0}\geq E,\\ {\hat{u}}_{0}{\hat{v}}_{0} & {\hat{u}}_{0}{\hat{v}}_{0}<E. \end{array}\right. \end{array}$$

If E=0, we can see from Figure 4c that $k^{0++}\left(E,{\hat{u}}_{0},{\hat{v}}_{0}\right)=k^{0--}\left(E,{\hat{u}}_{0},{\hat{v}}_{0}\right)={\hat{u}}_{0}{\hat{v}}_{0}$ and $k^{0-+}\left(E,{\hat{u}}_{0},{\hat{v}}_{0}\right)=k^{0+-}\left(E,{\hat{u}}_{0},{\hat{v}}_{0}\right)=0$.

The function value $k(E,{\hat{u}}_{0},{\hat{v}}_{0})$ in different cases is listed in Table 1. The symbol “∗” can be either “+”, “−”, or “0”, implying the positive or the negative sign of ${\hat{u}}_{0}$ or ${\hat{v}}_{0}$.

In the coordinate system $\left(\hat{u},\hat{v}\right)$, the original coordinates of four vertexes are converted into:

(28) $$\begin{array}{cc} \hat{A}= & \left({\hat{u}}_{A},{\hat{v}}_{A}\right)=\left(\frac{a_{01}}{a_{11}},\frac{a_{10}}{a_{11}}\right), \end{array}$$

(29) $$\begin{array}{cc} \hat{B}= & \left({\hat{u}}_{B},{\hat{v}}_{B}\right)=\left(1+\frac{a_{01}}{a_{11}},\frac{a_{10}}{a_{11}}\right), \end{array}$$

(30) $$\begin{array}{cc} \hat{C}= & \left({\hat{u}}_{C},{\hat{v}}_{C}\right)=\left(1+\frac{a_{01}}{a_{11}},1+\frac{a_{10}}{a_{11}}\right), \end{array}$$

(31) $$\begin{array}{cc} \hat{D}= & \left({\hat{u}}_{D},{\hat{v}}_{D}\right)=\left(\frac{a_{01}}{a_{11}},1+\frac{a_{10}}{a_{11}}\right). \end{array}$$

For similarity, let us define $H_{E}\left(P\right)≜k\left(E,u_{P},v_{P}\right)$. When considering various practical situations, no matter where the square *S* lies, the above-threshold area $|{\hat{S}}_{T}|$ is always provided by:

(32) $$\begin{array}{cc} \left|{\hat{S}}_{T}\right|= & \left|\int_{{\hat{u}}_{A}}^{{\hat{u}}_{C}}\int_{{\hat{v}}_{A}}^{{\hat{v}}_{C}}{𝟙}_{G_{E}}\left(\hat{u},\hat{v}\right)\;d\hat{u}\;d\hat{v}\right|\\ = & \left|\left(\int_{{\hat{u}}_{A}}^{0}\int_{{\hat{v}}_{A}}^{0}+\int_{0}^{{\hat{u}}_{C}}\int_{0}^{{\hat{v}}_{C}}+\int_{{\hat{u}}_{A}}^{0}\int_{0}^{{\hat{v}}_{C}}+\int_{0}^{{\hat{u}}_{C}}\int_{{\hat{v}}_{A}}^{0}\right){𝟙}_{G_{E}}\left(\hat{u},\hat{v}\right)d\hat{u}d\hat{v}\right|\\ = & \left|\int_{0}^{{\hat{u}}_{A}}\int_{0}^{{\hat{v}}_{A}}{𝟙}_{G_{E}}\left(\hat{u},\hat{v}\right)d\hat{u}d\hat{v}+\int_{0}^{{\hat{u}}_{C}}\int_{0}^{{\hat{v}}_{C}}{𝟙}_{G_{E}}\left(\hat{u},\hat{v}\right)d\hat{u}d\hat{v}\right.\\ & -\left.\int_{0}^{{\hat{u}}_{A}}\int_{0}^{{\hat{v}}_{C}}{𝟙}_{G_{E}}\left(\hat{u},\hat{v}\right)d\hat{u}d\hat{v}-\int_{0}^{{\hat{u}}_{C}}\int_{0}^{{\hat{v}}_{A}}{𝟙}_{G_{E}}\left(\hat{u},\hat{v}\right)d\hat{u}d\hat{v}\right|\\ = & \left|H_{E}\left(\hat{A}\right)-H_{E}\left(\hat{B}\right)+H_{E}\left(\hat{C}\right)-H_{E}\left(\hat{D}\right)\right|. \end{array}$$

When $a_{11}<0$, the above-threshold area $|{\hat{S}}_{T}|$ is the total area |S|, subtracting the area $|S_{T}|$ for $a_{11}>0$. We have:

(33) $$\left|{\hat{S}}_{T}\right|=1-\left|H_{E}\left(\hat{A}\right)-H_{E}\left(\hat{B}\right)+H_{E}\left(\hat{C}\right)-H_{E}\left(\hat{D}\right)\right|.$$

If $a_{11}=0$, the boundary is degraded into a straight line and can be transformed to:

(34) $$\hat{f}\left(u,v\right)=a_{10}u+a_{01}v+a_{00}=T.$$

Then, the above-threshold area $|{\hat{S}}_{T}|$ can be calculated according to the following four steps:

Step 1. If $\hat{f}\left(u_{A},v_{A}\right)\geq T$, add $A(u_{A},v_{A})$ to the ordered set *U*.

Step 2. If the edge $\bar{AB}$ is intersected with the boundary $\hat{f}\left(u,v\right)=T$ at the point $F(u_{F},v_{F})$ that satisfies $\hat{f}\left(u_{F},v_{F}\right)=T$, append $F(u_{F},v_{F})$ to the ordered set *U*.

Step 3. Similarly, judge if the point *B*, the intersection between $\bar{BC}$ and $\hat{f}\left(u,v\right)=T$, the point *C*, the intersection between $\bar{CD}$ and $\hat{f}\left(u,v\right)=T$, the point *D*, and the intersection between $\bar{DA}$ and $\hat{f}\left(u,v\right)=T$ should be added to the ordered set *U* according to the principles in Step 1 and Step 2.

Step 4. If ordered set U=⌀, $|{\hat{S}}_{T}|=0$. Otherwise, $|{\hat{S}}_{T}|$ is equal to the area of the polygon of which the vertexes are listed in the ordered set *U*.

So far, the above-threshold area $|{\hat{S}}_{T}|$ inside the subregion *S* is obtained by the bilinear interpolation for all the cases.

We propose a bilinear interpolation-based algorithm to estimate the above-threshold ratio in a predefined region, as shown in Algorithm 1.**Algorithm 1 BIB algorithm.****Input:** Region *R*, threshold *T*, the four coordinates $\left(x_{A},y_{A}\right)$, $\left(x_{B},y_{B}\right)$, $\left(x_{C},y_{C}\right)$, and $\left(x_{D},y_{D}\right)$, and the four sensor values $f_{A}$, $f_{B}$, $f_{C}$, and $f_{D}$ of all $S_{i}$, the heights and the widths of all $S_{i}$**Output:**${\hat{C}}_{T}$1:**for**i←1,m**do**2: $S\leftarrow S_{i}$.3: Calculate $a_{11}$, $a_{10}$, $a_{01}$, and $a_{00}$ with reference to *S*.4: **if**
$a_{11}\neq 0$
**then**5:  $\hat{u}\leftarrow u+\frac{a_{01}}{a_{11}}$6:  $\hat{v}\leftarrow v+\frac{a_{10}}{a_{11}}$7:  $Q\leftarrow \frac{a_{00}a_{11}-a_{01}a_{10}}{a_{11}}$8:  $E\leftarrow \frac{T-Q}{a_{11}}$9:  Calculate $H_{E}\left(\hat{A}\right)$, $H_{E}\left(\hat{B}\right)$, $H_{E}\left(\hat{C}\right)$, $H_{E}\left(\hat{D}\right)$.10:  $S^{\prime }\leftarrow H_{E}\left(\hat{A}\right)-H_{E}\left(\hat{B}\right)+H_{E}\left(\hat{C}\right)-H_{E}\left(\hat{D}\right)$.11:  **if**
$a_{11}>0$
**then**12:   $\left|{\hat{S}}_{T}\right|\leftarrow W\cdot H\cdot \left|S^{\prime }\right|$13:  **else**14:   $\left|{\hat{S}}_{T}\right|\leftarrow W\cdot H\cdot \left(1-\left|S^{\prime }\right|\right)$15:  **end if**16: **else**17:  U←{}18:  **for**
$j\leftarrow [A,\bar{AB},B,\bar{BC},C,\bar{CD},D,\bar{DA}]$
**do**19:   **if**
*j* in [A,B,C,D]
**then**20:    **if**
$\hat{f}\left(u_{j},v_{j}\right)\geq T$
**then**21:     Add *j* to *U*.22:    **end if**23:   **end if**24:   **if**
*j* in $\left[\bar{AB},\bar{BC},\bar{CD},\bar{DA}\right]$
**then**25:    **if**
*j* is intersected with the line $\hat{f}\left(u,v\right)=T$
**then**26:     Add the intersection to the ordered set *U*.27:    **end if**28:   **end if**29:  **end for**30:  **if**
U=⌀, $|{\hat{S}}_{T}|=0$
**then**31:   $|{\hat{S}}_{T}|\leftarrow 0$32:  **else**33:   $|{\hat{S}}_{T}|$ is equal to the area of the polygon of which the vertexes are listed in set *U*.34:  **end if**35: **end if**36: $\left|{\hat{S}}_{Ti}\right|\leftarrow \left|{\hat{S}}_{T}\right|$37:**end for**38:${\hat{C}}_{T}\leftarrow \frac{1}{\left|R\right|}\cdot \sum_{i=1}^{m}\left|{\hat{S}}_{Ti}\right|$

### 4.3. Nearest Neighbor Algorithm and Marching Square Algorithm

Both the NN and MS algorithm utilize the sensor values of four vertexes in the rectangle subregion.

In the NN algorithm, the sensor value of arbitrary coordinate (u,v) in subregion *S* is determined by that of the nearest vertex, and the interpolation weight $W_{\mathrm{NN}}\left(u,v\right)$ in Equation (8) can be expressed as:

(35) $$W_{\mathrm{NN}}\left(u,v\right)=\left(\begin{array}{c} {𝟙}_{\left[0,\frac{1}{2}\right]\times \left[0,\frac{1}{2}\right]}\left(u,v\right)\\ {𝟙}_{\left(\frac{1}{2},1\right]\times \left[0,\frac{1}{2}\right]}\left(u,v\right)\\ {𝟙}_{\left(\frac{1}{2},1\right]\times \left(\frac{1}{2},1\right]}\left(u,v\right)\\ {𝟙}_{\left[0,\frac{1}{2}\right]\times \left(\frac{1}{2},1\right]}\left(u,v\right) \end{array}\right).$$

**Figure 7:**
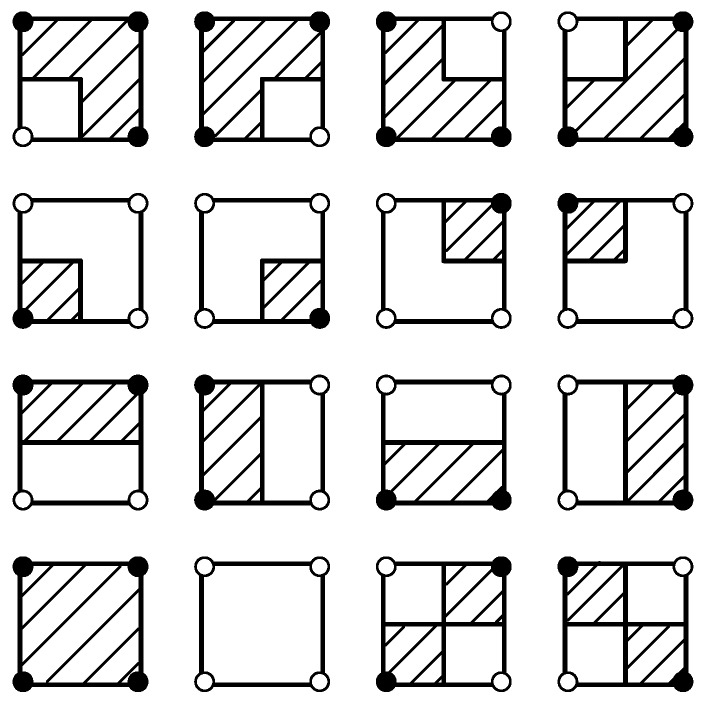
Illustrations of the boundaries and the above-threshold regions with the NN algorithm.

The illustrations of the boundaries and the above-threshold regions with the NN algorithm are shown in Figure 7.

**Figure 8:**
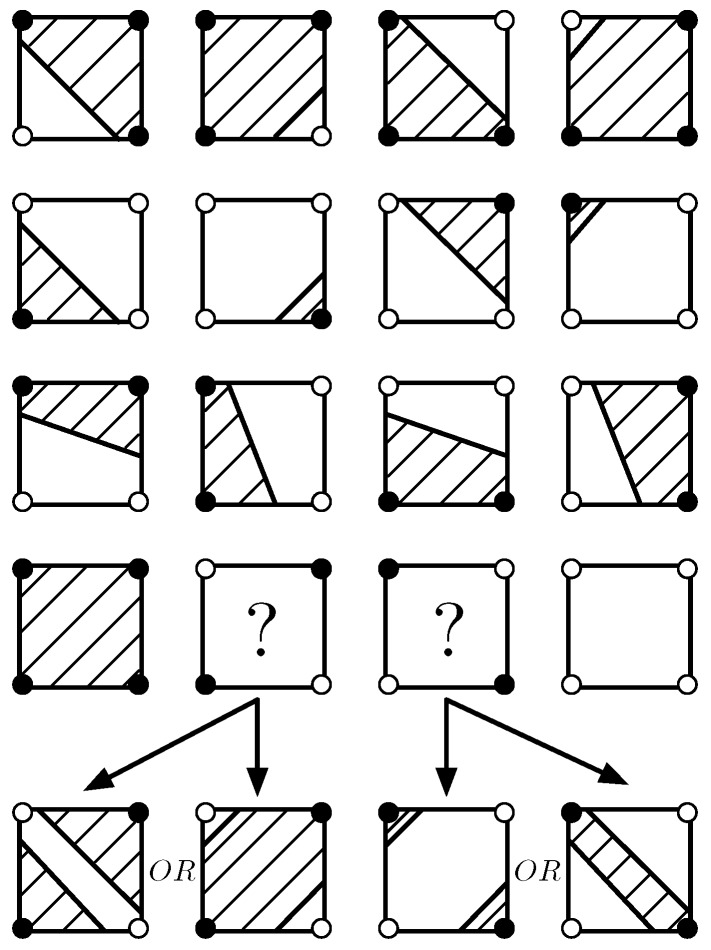
Illustrations of the boundaries and the above-threshold regions with the Marching Square (MS) algorithm.

The MS algorithm is a computer graphics algorithm, and thus, its interpolation weight does not exist. The illustrations of the boundaries and the above-threshold regions with the MS algorithm are depicted in Figure 8. Note that there are two ambiguous situations for above-threshold calculation and boundary description. The MS algorithm is possibly considered as an approximation of the BIB algorithm, which transforms the hyperbolic boundaries by the BIB algorithm into the straight lines.

Note that in order to estimate the above-threshold coverage ratio in region *R*, we sum the above-threshold area limited inside each rectangle grid $S_{i}$ for the three algorithms. Thus, the algorithm complexities of the three mentioned algorithms are both O(M), where *M* is the number of grids.

### 4.4. Evaluative Metrics

The MSE in mathematical statistics refers to the mean value of the squares of the differences between the estimated values and true values, which is given by:

(36) $$MSE=\frac{1}{N_{e}}\sum_{t=1}^{N_{e}}{\left(observed_{t}-predicted_{t}\right)}^{2}$$

where $observed_{t}$ represents the exact above-threshold ratio in the *t*th experiment, $predicted_{t}$ is the estimated above-threshold ratio obtained by different algorithms in the *t*th experiment, and $N_{e}$ is the number of experiments. A lesser MSE is better.

In statistical hypothesis testing, the FP and FN are known as type I and type II errors, where FP corresponds to the situation when the exact value is false, while the estimated value is true and FN is opposite. True Positive (TP) represents that the exact value and the estimated value are both true, and True Negative (TN) represents both false. In the above-threshold queries, the conditions of FP, FN, TP, and TN are listed in Table 2. The probability of FP and FN in a single experiment can be expressed as:

(37) $$\begin{array}{c} FP=\frac{1}{m}\sum_{i=1}^{m}\frac{\left|\left(S-S_{Ti}\right)\cap {\hat{S}}_{Ti}\right|}{\left|S\right|}, \end{array}$$

(38) $$\begin{array}{c} FN=\frac{1}{m}\sum_{t=1}^{m}\frac{|S_{Ti}\cap \left(S-{\hat{S}}_{Ti}\right)|}{\left|S\right|}. \end{array}$$

## 5. Experiments and Discussion

In this section, we first testify to the correctness of the proposed algorithm by theory analysis. Then, we describe the experiments with both the artificially-constructed environment data and the real temperature data. All programming and benchmarking were done in Python (Version 3.6) with the Numpy [39] and pyKrige [40] libraries running on a computer with a 2600-MHz Intel processor and 8-gigabyte memories. Furthermore, we illustrate the possible implementation frameworks in wireless sensor network and analyze the algorithm complexity. Finally, we provide the evaluation and the comparison of the NN, MS, and BIB algorithms.

### 5.1. Theory Analysis

In geography, the Kriging algorithm is one of the most wide-spread interpolation algorithms and can be regarded as the gold standard [41].

We use the local four-point Kriging (Kr4) interpolation to validate the performance of the proposed algorithm. Assume that the Kr4 interpolation is controlled by the four vertexes of the square. The semi-variable function is linear, and the nugget is zero. The interpolation weight $W_{\mathrm{Kr}4}\left(u,v\right)$ in Equation (8) can be calculated as:

(39) $$W_{\mathrm{Kr}4}\left(u,v\right)=\left(\begin{array}{c} a_{1}b_{1}+a_{2}b_{2}+a_{3}b_{3}+a_{2}b_{4}+\frac{1}{4}\\ a_{2}b_{1}+a_{1}b_{2}+a_{2}b_{3}+a_{3}b_{4}+\frac{1}{4}\\ a_{3}b_{1}+a_{2}b_{2}+a_{1}b_{3}+a_{2}b_{4}+\frac{1}{4}\\ a_{2}b_{1}+a_{3}b_{2}+a_{2}b_{3}+a_{1}b_{4}+\frac{1}{4} \end{array}\right)$$

where:

(40) $$\begin{array}{cc} a_{1}= & -\frac{1}{4}-\frac{3\sqrt{2}}{8},\;a_{2}=\frac{1}{4}+\frac{\sqrt{2}}{8},\;a_{3}=-\frac{1}{4}+\frac{\sqrt{2}}{8},\\ b_{1}= & \sqrt{u^{2}+v^{2}},\;b_{2}=\sqrt{{\left(u-1\right)}^{2}+v^{2}},\\ b_{3}= & \sqrt{{\left(u-1\right)}^{2}+{\left(v-1\right)}^{2}},\;b_{4}=\sqrt{u^{2}+{\left(v-1\right)}^{2}}. \end{array}$$

**Figure 9:**
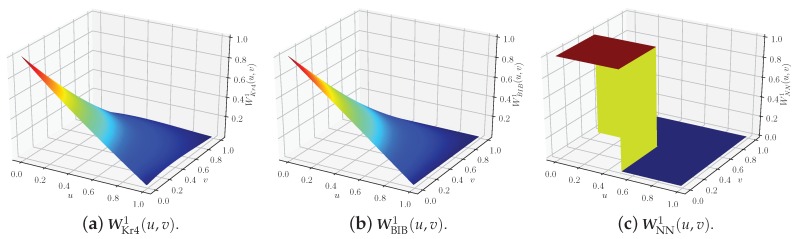
Function images of $W_{\mathrm{Kr}4}^{1}\left(u,v\right)$, $W_{\mathrm{BIB}}^{1}\left(u,v\right)$, and $W_{\mathrm{NN}}^{1}\left(u,v\right)$.

We denote the *i*th elements in $W_{\mathrm{Kr}4}\left(u,v\right)$, $W_{\mathrm{BIB}}\left(u,v\right)$ and $W_{\mathrm{NN}}\left(u,v\right)$ as $W_{\mathrm{Kr}4}^{i}\left(u,v\right)$, $W_{\mathrm{BIB}}^{i}\left(u,v\right)$, and $W_{\mathrm{NN}}^{i}\left(u,v\right)$, respectively, and these values are shown in Figure 9 for i=1. We can conclude that the weights $W_{\mathrm{Kr}4}^{1}\left(u,v\right)$ and $W_{\mathrm{BIB}}^{1}\left(u,v\right)$ are almost identical, while the weight $W_{\mathrm{NN}}^{1}\left(u,v\right)$ is quite different from the other two.

**Figure 10:**
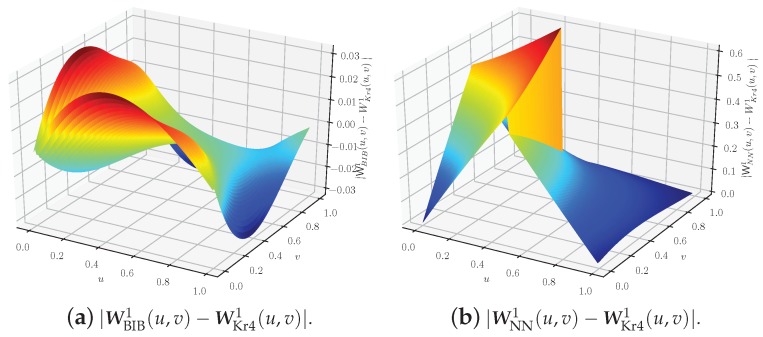
Function images of $|W_{\mathrm{BIB}}^{1}\left(u,v\right)-W_{\mathrm{Kr}4}^{1}\left(u,v\right)|$ and $|W_{\mathrm{NN}}^{1}\left(u,v\right)-W_{\mathrm{Kr}4}^{1}\left(u,v\right)|$.

The differences of weights, i.e., $|W_{\mathrm{BIB}}^{1}\left(u,v\right)-W_{\mathrm{Kr}4}^{1}\left(u,v\right)|$ and $|W_{\mathrm{NN}}^{1}\left(u,v\right)-W_{\mathrm{Kr}4}^{1}\left(u,v\right)|$, are shown in Figure 10. The largest weight error $\max_{u,v}\left|W_{\mathrm{BIB}}^{1}\left(u,v\right)-W_{\mathrm{Kr}4}^{1}\left(u,v\right)\right|$ is less than 0.03 in the BIB algorithm, while $\max_{u,v}\left|W_{\mathrm{NN}}^{1}\left(u,v\right)-W_{\mathrm{Kr}4}^{1}\left(u,v\right)\right|$ is more than 0.5 in the NN algorithm. The same conclusion holds for the other weights $W_{\mathrm{Kr}4}^{i}\left(u,v\right)$, $W_{\mathrm{BIB}}^{i}\left(u,v\right)$, and $W_{\mathrm{NN}}^{i}\left(u,v\right)$, i=2,3,4. Therefore, the interpolation result of the BIB algorithm almost approximates that of Kr4 algorithm. Note that the above-threshold area is difficult to be obtained by the Kr4 algorithm due to the complex calculation of the integral inside a complex boundary. Moreover, the NN algorithm has a large weight error in geographic data analysis.

### 5.2. Experiment on Artificially-Constructed Environment Data

In order to evaluate different above-threshold query algorithms, we test them with artificial data generated by the Kriging algorithm. The interpolated values by the Kriging algorithm are modeled as a Gaussian process governed by full prior covariances.

**Figure 11:**
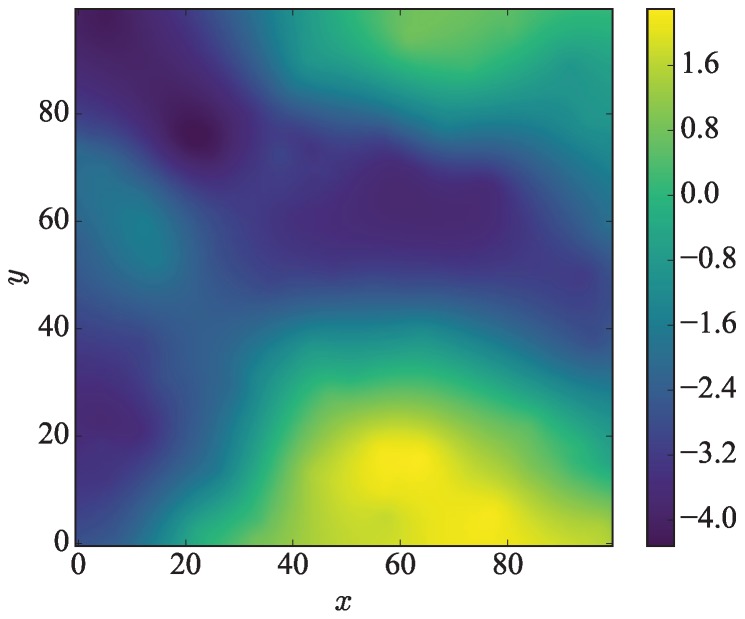
Artificial environmental variables for simulation experiments.

Assume that the Kriging interpolation is controlled by $N_{c}$ points $P_{i}$, $1\leq i\leq N_{c}$, randomly distributed in *R*, and the value *f* at $P_{i}$ is an independent random variable uniformly distributed in the interval [a,b]. Typical artificial environmental variables are generated by $N_{c}$ control points inside a 300×300 region. We denote the central 100×100 region as the ROI in which the sensor values are depicted in Figure 11 in a single experiment. The greater the number of the control points is, the richer the details of the variances of artificial environmental variables are.

To compare different algorithms with the same number of sensors, we assume that there are in total N=(n+1)×(n+1) sensors and M=n×n grids.

We performed simulation experiments to contrast the three algorithms, NN, MS, and BIB, to estimate the above-threshold ratios. The simulation configuration is given in Table 3.

**Figure 12:**
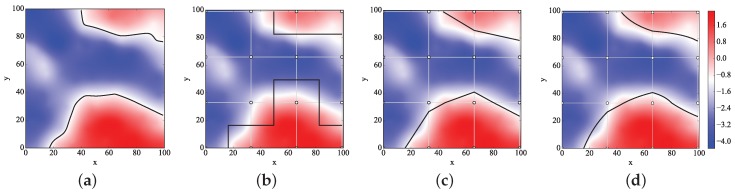
The real boundary and the boundaries generated by the three mentioned algorithms in ROI when n=3 in the artificially-constructed environment data experiment. (**a**) The real boundary. (**b**) The boundaries by the NN algorithm. (**c**) The boundaries by the MS algorithm. (**d**) The boundaries by the BIB algorithm.

The real boundary is shown in Figure 12a, and the boundaries generated by the three algorithms in ROI when n=3 are drawn in Figure 12b–d. Obviously, the boundaries by the BIB and MS algorithms are more accurate than those by the NN algorithm. Furthermore, the boundaries by the BIB algorithm are smoother than those by the other algorithms.

**Figure 13:**
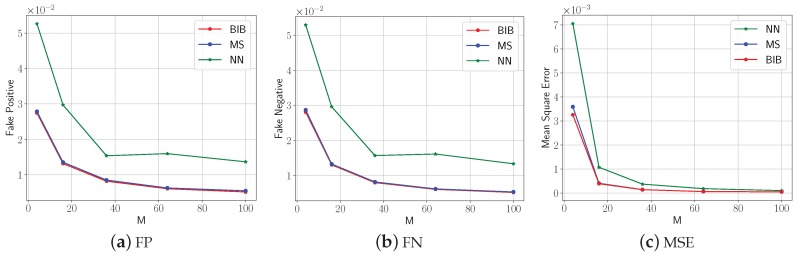
The average FP, FN, and MSE versus the grid numbers *M* for the three mentioned algorithms in the artificially-constructed environment data experiment.

In order to analyze the three aforementioned algorithms qualitatively, we used the metrics FP, FN, and MSE to evaluate their performance for $N_{e}$ experiments. The average FP, FN, and MSE versus the number of grids *M* for the three mentioned algorithms are shown in Figure 13.

The threshold $T=\frac{f_{\max}+f_{\min}}{2}$, where $f_{\max}$ and $f_{\min}$ are the maximum and the minimum of the variables. First, all the FP, FN, and MSE metrics obtained by the three algorithms tend to a definite value, zero, along with the increasing of the number of sensors. This implies all algorithms are feasible and more sensors help to reduce the query error. Second, the NN algorithm performs worse than the others. This is mainly caused by the rough estimating of the variables inside the ROI. Since the proposed BIB algorithm is an approximation of the Kr4 algorithm and the MS algorithm is an approximation of the BIB algorithm, the MS and BIB algorithms both performed well.

### 5.3. Experiment of Real Temperature Data

WorldClim [42] is a set of gridded global climate data with a spatial resolution from 1–340 km${}^{2}$ over 30 years, which was created by Steve Fick and Robert Hijmans. WorldClim provides various environmental variables, including minimum temperature (${}^{\circ }C$), maximum temperature (${}^{\circ }C$), average temperature (${}^{\circ }C$), precipitation (mm), solar radiation ($\mathrm{kJ}m^{-2}{\mathrm{day}}^{-1}$), wind speed ($ms^{-1}$) and water vapor pressure (kPa). These data can be used for mapping and spatial modeling.

**Figure 14:**
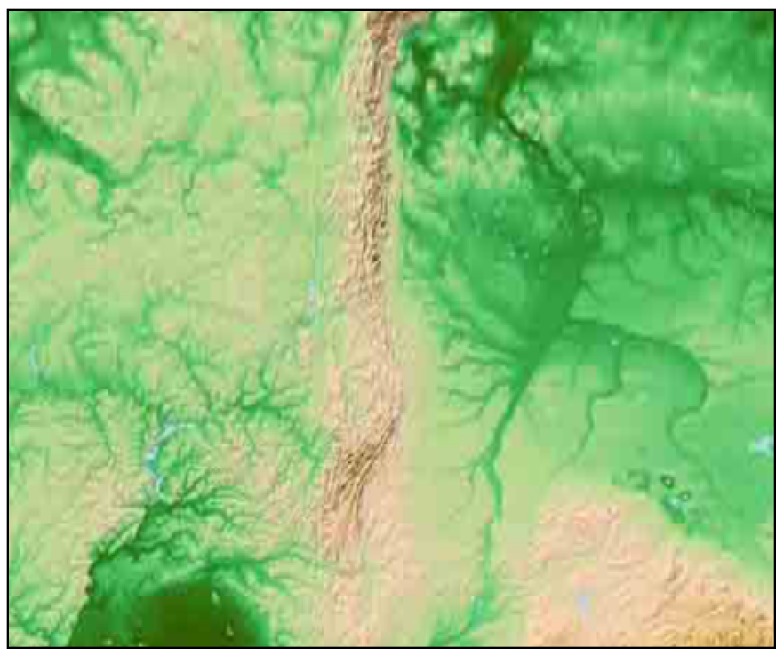
The hypsometric map of the ROI.

The global average monthly temperature data, that is 12 sets of temperature data, obtained by WorldClim from 1970–2000, were used in the following experiments. We first randomly selected an ROI that did not contain a water surface, ranging from 42–$78^{\circ }$ E and from 49–$65^{\circ }$ N, to observe the boundaries. The hypsometric map of the ROI is depicted in Figure 14.

**Figure 15:**
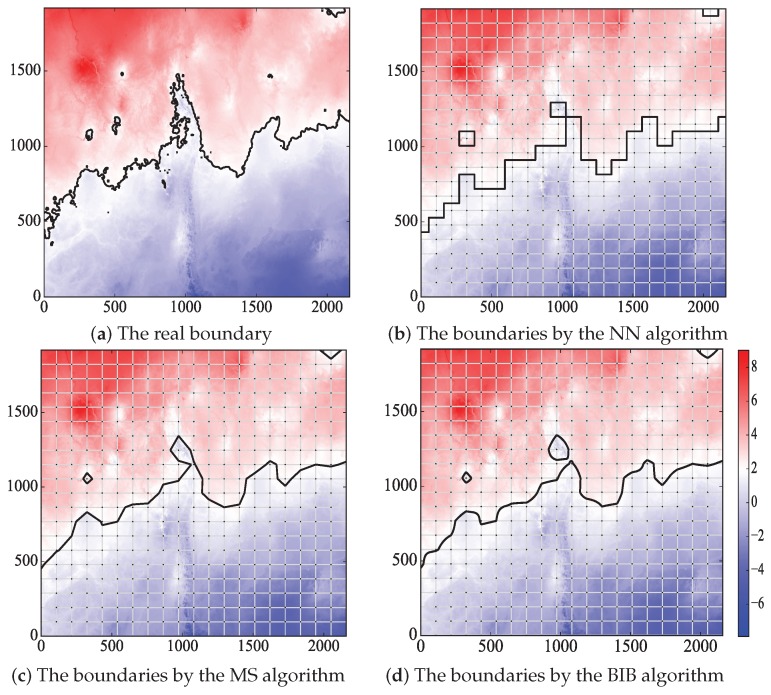
The real boundary and the boundaries generated by the three mentioned algorithms in ROI when n=20 in the real temperature data experiment.

Rectangle divisions were done to the ROI, and M=n×n denotes the grid numbers. The real boundary and the boundaries generated by the three algorithms in ROI when n=20 are depicted in Figure 15.

Obviously, the boundaries by the BIB algorithm are more accurate than those by the NN and MS algorithms. Furthermore, the boundaries by the BIB algorithm are smoother than those by the other algorithms.

**Figure 16:**
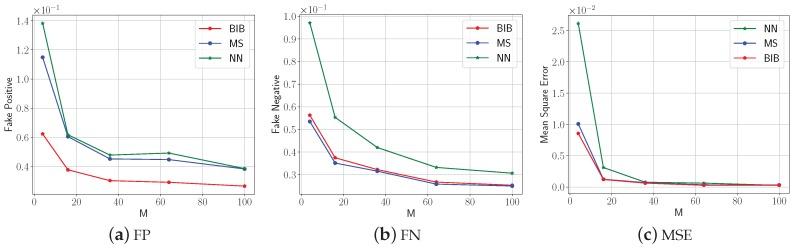
The average FP, FN, and MSE versus the grid numbers *M* for the three mentioned algorithms in the real temperature data experiment.

Then, we randomly selected 20 ROIs that did not contain a water surface with a length of $10^{\circ }$ both in the latitude and longitude direction in Eurasia to demonstrate the universality of the proposed algorithm. We assumed that in the case that the distance between the sampling points was 30 s, i.e., the most precise spatial resolution, the average above-threshold ratio obtained by the NN algorithm was the exact above-threshold ratio. The average FP, FN, and MSE versus the number of grids *M* for the three mentioned algorithms in 20 ROIs are shown in Figure 16. The threshold *T* was assigned to the median of the temperature data. Although the effects of the results cannot be favorably compared with those in the artificial experiment due to the discontinuity points such as the mountain segregation, the shapes and the trends of the curves in the real temperature data experiment are the same as those in the artificially-constructed environment data experiment. Above all, the proposed BIB algorithm shows a good performance in estimating the above-threshold ratio and solving the above-threshold query equation within the given ROI.

### 5.4. Possible Implementation Framework and Computation/Communication Load Analysis

**Figure 17:**
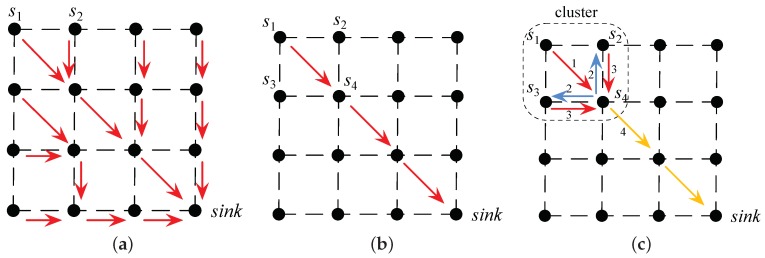
Possible implementation frameworks in wireless sensor networks. (**a**) Centralized query framework. (**b**) Distributed implementation framework for the NN algorithm. (**c**) Distributed implementation framework for the MS or BIB algorithm.

A naive implementation framework to support the aforementioned above-threshold queries with the BIB, NN, or MS algorithms is to collect all the sensor data and then process the data in the center server. All the positions of the nodes were assumed to be obtained with node location techniques. A possible data collection route tree to transfer the sensor data from nodes is shown in Figure 17a. In such a naive implementation, the nodes only send out their data, and the center server needs to execute the query, which is thus named the centralized query framework. The traffic loads in the sensor network of different algorithms were equal. However, the center server needed more computation for the BIB and MS algorithms to perform the interpolation than that for the NN algorithm, which in fact simply counted the number of the data above the threshold.

However, in most practical scenarios, there would be only a small proportion of the sensor values above the threshold. We herein propose a distributed implementation framework to support the aforementioned above-threshold queries.

In this distributed implementation framework to support the NN algorithm, the sensor will transfer its sensor value if and only if it is triggered by the above-threshold event, i.e., its sensor value is beyond the threshold, as shown in Figure 17b. The nodes in the route path from the triggered node to the sink node help to forward the data hop by hop. Therefore, the center server only counted the data from the triggered nodes, and the values of other nodes were assumed to be below the threshold. Now, the network traffic was significantly reduced if the above-threshold events occurred sparsely.

It is more complicated to support the MS and BIB algorithms since both need local co-operation. We first grouped the sensor nodes into overlapped clusters. All four nodes in the vertexes of a square grid make a cluster. One node possibly belongs to a different cluster since it may be shared by neighbor grids. There was a cluster head for each cluster, as shown in Figure 17c. When a node, say, $s_{1}$, is triggered by the above-threshold event, it will send its sensor value to notify the cluster heads of all the clusters to which it belongs. Each cluster head notified, say $s_{4}$, will command its cluster members to submit their sensor values to it. Once the header collects all the sensor values in the cluster, it will perform the computation and transfer the fused result to the sink hop by hop. Therefore, the MS and BIB algorithms needed more local traffic and more computation to exchange their sensor values for the interpolation.

### 5.5. Comparison and Discussion

We evaluate and compare the three mentioned algorithms, NN, MS, and BIB, according to various evaluative metrics in Table 4. We can see from Figure 12 and Figure 15 that the curve boundaries by the BIB algorithm are more accurate and smoother than the straight line boundaries by the other two algorithms. We can see from Figure 13 and Figure 16 that all algorithms are feasible and that NN algorithm performed worse than the others. The largest weight error is less than 0.03 in the BIB algorithm, while it is more than 0.5 in the NN algorithm. Therefore, the interpolation result of the BIB algorithm almost approximates that of the Kr4 algorithm. Since the MS algorithm is possibly considered as an approximation of the BIB algorithm, the same conclusion holds for it. Both the BIB and the MS algorithms have small weight errors in geographic data analysis, while the NN algorithm have the larger one. The interpolation weights $W_{BIB}\left(u,v\right)$ and $W_{NN}\left(u,v\right)$ are given by Equations (9) and (35), respectively. The MS algorithm is a computer graphics algorithm, and thus, its interpolation weight does not exist. We can see from Figure 8 that there were two ambiguous situations for above-threshold area calculation and boundary description in the MS algorithm. For the distributed implementation framework, once the header collects all the sensor values in the cluster, it will perform the computation and transfer the fused result to the sink hop by hop in the MS and BIB algorithms. The center server only counts the data from the triggered nodes and the values of other nodes were assumed to be below the threshold in NN algorithm. Therefore, the MS and BIB algorithms need more local traffic and more computation to exchange their sensor values for the interpolation. The traffic and computation are modest in the MS and BIB algorithms due to the small above-threshold clusters. Taking all the factors into consideration, the proposed BIB algorithm has an excellent comprehensive performance compared with the other algorithms.

## 6. Conclusions

Wireless sensor networks can be regarded as sensor database systems, which permit users to query sensor data of interest. In this paper, we proposed a novel Bilinear Interpolation-Based (BIB) algorithm to support the above-threshold queries. It is mainly designed for regularly-deployed sensor networks. We utilize the bilinear interpolation to estimate the environmental variables inside a square with the known sensor values from sensor nodes located at its four vertexes. Thus, the area of the above-threshold subregion can be obtained by mathematical formulation, and finally, we proposed the closed-form solution of the above-threshold ratio.

In order to evaluate the performance of different above-threshold query algorithms, we first used the interpolation weights of Kr4 interpolation, which is commonly used in geometry research, as a golden standard to compare various algorithms. The weights of the proposed BIB algorithm are approximate to those of the Kr4 interpolation, which means BIB is valid from the perspective of geography.

We also described experiments both with the artificially-constructed environment data and with real temperature data. Experiment results manifest that the proposed BIB algorithm shows a good performance in estimating the above-threshold ratio.

In summary, with a closed-form formulation, the proposed BIB algorithm supports the above-threshold queries in an efficient and accurate manner.

## Figures and Tables

**Table 1:** The function value $k(E,{\hat{u}}_{0},{\hat{v}}_{0})$ in different cases.

*E*	${\hat{u}}_{0}$	${\hat{v}}_{0}$	$k(E,{\hat{u}}_{0},{\hat{v}}_{0})$
+	+	+	$\left\{\begin{array}{cc} {\hat{u}}_{0}{\hat{v}}_{0}-E-Eln\frac{{\hat{u}}_{0}{\hat{v}}_{0}}{E} & \left({\hat{u}}_{0}{\hat{v}}_{0}\geq E\right)\\ 0 & \left({\hat{u}}_{0}{\hat{v}}_{0}<E\right) \end{array}\right.$
+	−	+	0
+	−	−	$\left\{\begin{array}{cc} {\hat{u}}_{0}{\hat{v}}_{0}-E-Eln\frac{{\hat{u}}_{0}{\hat{v}}_{0}}{E} & \left({\hat{u}}_{0}{\hat{v}}_{0}\geq E\right)\\ 0 & \left({\hat{u}}_{0}{\hat{v}}_{0}<E\right) \end{array}\right.$
+	+	−	0
+	0	∗	0
+	∗	0	0
−	+	+	${\hat{u}}_{0}{\hat{v}}_{0}$
−	−	+	$\left\{\begin{array}{cc} -E-Eln\frac{{\hat{u}}_{0}{\hat{v}}_{0}}{E} & \left({\hat{u}}_{0}{\hat{v}}_{0}\geq E\right)\\ {\hat{u}}_{0}{\hat{v}}_{0} & \left({\hat{u}}_{0}{\hat{v}}_{0}<E\right) \end{array}\right.$
−	−	−	${\hat{u}}_{0}{\hat{v}}_{0}$
−	+	−	$\left\{\begin{array}{cc} -E-Eln\frac{{\hat{u}}_{0}{\hat{v}}_{0}}{E} & \left({\hat{u}}_{0}{\hat{v}}_{0}\geq E\right)\\ {\hat{u}}_{0}{\hat{v}}_{0} & \left({\hat{u}}_{0}{\hat{v}}_{0}<E\right) \end{array}\right.$
−	0	∗	0
−	∗	0	0
0	+	+	${\hat{u}}_{0}{\hat{v}}_{0}$
0	−	+	0
0	−	−	${\hat{u}}_{0}{\hat{v}}_{0}$
0	+	−	0
0	0	∗	0
0	∗	0	0

**Table 2:** False positive, false negative, true positive, and true negative.

	Estimated Sensor Value	Positive (Above T)	Negative (Under T)
Exact Sensor Value			
Positive (Above T)		TP	FN
Negative (Under T)		FP	TN

**Table 3:** Simulation configuration.

Parameter	Value
Side length of *R*	100
$N_{c}$	100
*a*	−20
*b*	20
$N_{e}$	2000

**Table 4:** Algorithm comparison.

	Algorithm	NN	MS	BIB
Index				
Boundary Accuracy		Worse	Good	Best
Boundary Continuity		Worse	Good	Best
FP		Worse	Good	Good
FN		Worse	Good	Good
MSE		Worse	Good	Good
Geographic Interpretation		Worse	Good	Good
Closed-form Solution		Yes	No	Yes
Ambiguity		No	Sometimes	No
Computation Workload		Little	Medium	Medium
Communication Traffic		Little	Medium	Medium
